# Digital Validation in Breast Cancer Needle Biopsies: Comparison of Histological Grade and Biomarker Expression Assessment Using Conventional Light Microscopy, Whole Slide Imaging, and Digital Image Analysis

**DOI:** 10.3390/jpm14030312

**Published:** 2024-03-16

**Authors:** Ji Eun Choi, Kyung-Hee Kim, Younju Lee, Dong-Wook Kang

**Affiliations:** 1 Department of Pathology, Chungnam National University Sejong Hospital, 20 Bodeum 7-ro, Sejong 30099, Republic of Korea; 2 Department of Pathology, Chungnam National University School of Medicine, 266 Munhwa Street, Daejeon 35015, Republic of Korea; phone330@cnu.ac.kr; 3 Department of Surgery, Chungnam National University Sejong Hospital, 20 Bodeum 7-ro, Sejong 30099, Republic of Korea; jaennejuya@cnuh.co.kr

**Keywords:** breast cancer, core needle biopsy, histological grade, biomarkers, validation, whole slide imaging, digital image analysis

## Abstract

Given the widespread use of whole slide imaging (WSI) for primary pathological diagnosis, we evaluated its utility in assessing histological grade and biomarker expression (ER, PR, HER2, and Ki67) compared to conventional light microscopy (CLM). In addition, we explored the utility of digital image analysis (DIA) for assessing biomarker expression. Three breast pathologists assessed the Nottingham combined histological grade, its components, and biomarker expression through the immunohistochemistry of core needle biopsy samples obtained from 101 patients with breast cancer using CLM, WSI, and DIA. There was no significant difference in variance between the WSI and CLM agreement rates for the Nottingham grade and its components and biomarker expression. Nuclear pleomorphism emerged as the most variable histologic component in intra- and inter-observer agreement (kappa ≤ 0.577 and kappa ≤ 0.394, respectively). The assessment of biomarker expression using DIA achieved an enhanced kappa compared to the inter-observer agreement. Compared to each observer’s assessment, DIA exhibited an improved kappa coefficient for the expression of most biomarkers with CLM and WSI. Using WSI to assess prognostic and predictive factors, including histological grade and biomarker expression in breast cancer, is acceptable. Furthermore, incorporating DIA to assess biomarker expression shows promise for substantially enhancing scoring reproducibility.

## 1. Introduction

The assessment of breast cancer (BC) histological grade and biomarker expression has become routine practice in clinical pathology. The histological grading of BC is one of the strongest prognostic factors and has been included in the American Joint Committee on Cancer (AJCC) staging system as a stage modifier [1]. Beyond its role as a prognostic factor, histological grading is also essential for recognizing when the histological grade of BC is unusual or discordant with hormone receptor or human epidermal growth factor receptor 2 (HER2) status; further work-up is warranted to ensure accurate histological typing, grade, and biomarker status [2]. BC biomarkers, including the estrogen receptor (ER), progesterone receptor (PR), HER2, and Ki67, are well-established prognostic factors that play crucial roles in determining biological subtypes and guiding therapeutic strategies for patients [3,4]. Hence, there is a substantial demand for accurate, precise, and standardized evaluation of these biomarkers. ER, PR, HER2, and Ki67 analyzed through immunohistochemistry (IHC) could act as surrogate markers for gene expression-based subtypes to reflect prognosis. Such assays are generally more accessible than gene expression molecular profiling assays, which are costly and time-consuming [5]. The interpretation of BC biomarkers through IHC is a critical component of pathological reporting, especially since the St. Gallen consensus guidelines endorsed it as a diagnostic standard [6].

Digital pathology, originally known as “telepathology”, has seen significant progress since its advent in the 1980s. Digital imaging hardware and software innovations have led to whole slide imaging (WSI), in which glass slides of pathological specimens are digitally scanned at a high resolution for viewing on a computer screen [7,8]. Recently, WSI has been used globally for digital imaging preservation, education, teleconsultation, and, increasingly, primary pathological diagnosis because it has several advantages over conventional light microscopy (CLM), such as portability, ease of sharing and retrieval of archival images, and the ability to utilize computer-aided diagnostic tools [9,10,11]. When using WSI for practical diagnostic purposes, validating specific WSI systems before clinical use is necessary to ensure accurate diagnoses to at least the same level as CLM [12,13]. Studies validating WSI systems for primary diagnostic purposes have been conducted by pathology laboratories across various subspecialties, including breast pathology [14,15,16]. However, most of these studies primarily used hematoxylin–eosin (H&E) slides for primary diagnosis rather than focusing on the assessment of prognostic pathologic factors or the expression of IHC-stained biomarkers. As WSI becomes the established norm in surgical pathology, a pertinent line of inquiry emerges concerning the potential influence of integrating digital pathology into patient prognostic indicators within a real-world clinical environment. Consistently, there have been concerns regarding the use of WSI as a primary diagnostic tool in breast pathology, including the assessment of prognostic and predictive variables such as histological grade determination and interpretation of biomarker staining results [7,17,18]. Furthermore, given the inherent limitations of visual assessment for evaluating biomarker expression using IHC, automated digital image analysis (DIA) has been proposed as a potential method to improve accuracy and inter-observer reproducibility when assessing IHC expression, and its utility has been analyzed [17,19].

Core needle biopsy (CNB) is one of the most common methods for performing pathological breast lesion diagnosis [20]. When diagnosing invasive BC through CNB, it is imperative to assess not only the initial histological grade but also ER, PR, and HER2 through IHC testing [21]. This process informs critical treatment decisions regarding potential neoadjuvant therapy before surgical interventions. In instances of complete pathological responses, the biopsy sample represents the sole remains of the available tumor [22]. Additionally, CNB samples are preferred over excision for biomarker testing because this approach helps to prevent many fixation problems [3,23]. Consequently, ensuring reliable evaluation of the histological grade and BC biomarker expression in CNB samples is critical, whether using CLM or WSI.

This study aimed to evaluate the effectiveness and reliability of WSI in BC CNB as a primary diagnostic method, focusing on determining the histological grade and characterizing biomarker expression, compared with CLM systems. The feasibility of using DIA to assess biomarker expression in clinical practice was also evaluated.

## 2. Materials and Methods

### 2.1. Case Selection and Immunohistochemistry

A total of 115 specimens of primary BC cases previously diagnosed through US-guided CNB at Chungnam National University Sejong Hospital from July 2020 to December 2022 were retrospectively analyzed. All routine H&E-stained slides were collected and reviewed. Specimens with scant tumor cells or poor fixation for IHC staining were excluded (*n* = 14). Four 4 μm sections from each formalin-fixed paraffin-embedded block were subjected to IHC using the Dako Omnis autostaining device (Agilent Technologies, Santa Clara, CA, USA). Four primary antibodies were used: ER (1:100, 6F11; Novocastra Laboratories, Newcastle, UK), PR (1:100, 16; Novocastra Laboratories), HER2 (C-erbB2 oncoprotein, 1:600, polyclonal; Dako, Glostrup, Denmark), and Ki-67 (1:50, MIB-1; Dako). This study was approved by the Institutional Review Board of Chungnam National University Sejong Hospital (IRB No. 2022-10-005) and contained a waiver for written informed consent based on the retrospective and anonymous character of this study.

### 2.2. Conventional Light Microscope and Pathologist Visual Grading and Scoring

**Figure 1:**
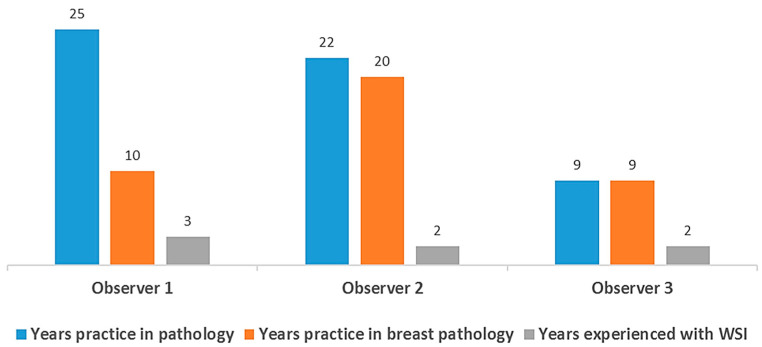
Three board-certified pathologists participated in this study, and their years of experience were documented. WSI, whole slide imaging.

Histological grading and IHC staining assessment were performed using glass slides via CLM with eyepieces with a field number of 22 mm (Nikon Ci-L, Tokyo, Japan). Cases were initially reviewed by three board-certified pathologists, each with varying levels of experience and training (Figure 1). The Nottingham combined histological grade (NG; Nottingham modification of the Scarff–Bloom–Richardson grading system) is recommended for the histological grading of conventional H&E slides by the College of American Pathologists and WHO guidelines. For this grade, the score for three categories is totaled: tubule formation (TF) as an expression of glandular differentiation (score 1–3), nuclear pleomorphism (NP) (score 1–3), and mitotic counts (MCs) (score 1–3). Combined scores of 3–5, 6–7, and 8–9 points were classified as grades 1, 2, and 3, respectively [24]. The interpretation of ER and PR was based on the Allred score and defined positive when ≥1% of the tumor cell nuclei showed immunostaining, according to the 2010 ASCO/CAP guidelines [23,25]. HER2 IHC was regarded as negative (0 or 1+), equivocal (2+), or positive (3+) based on the 2018 ASCO/CAP guidelines [26]. Nuclear staining of any intensity was defined as Ki67 positive. The assessment of Ki67 staining was conducted globally by determining an average score across all tumor cells in invasive tumor areas, scored as 0, 1 (≤5%), 2 (5–30%), and 3 (≥30%) based on the report of the International Working Group on Ki67 in Breast Cancer [3]. The assessment of biomarkers’ IHC expression scores was conducted through a consensus meeting involving three observers to compare DIA results.

### 2.3. Slide Digitization, Re-Grading, and Scoring with WSI

For WSI, H&E and corresponding IHC-stained slides were imaged at a high resolution (0.121 µm/pixel) and 40× magnification (40×/0.95 Plan-Apochromat, Carl Zeiss Microscopy, NY, USA) with a single z-plane using a whole slide scanner (PANNORAMIC 250 Flash III, 3DHISTECH, Budapest, Hungary). Digital images were generated and saved in the MRXS format, managed with server software (Panoramic Scanner, 3DHISTECH), and retrieved using a file management web interface (CaseViewer, 3DHISTECH). The mean file size of the scanned images was as follows: 1.95 GB for H&E, 1.24 GB for ER, 1.24 GB for PR, 1.50 GB for HER2, and 1.39 GB for Ki67. Scanned digital images were evaluated for quality to ensure that they were in focus and analyzed using 27-inch 3840 × 2160 resolution monitors (4 K UHD, LG, Seoul, Korea).

For the intra-observer agreement of BC histological grading using WSI, all three pathologists graded all included cases using WSI blinded to the CLM grade and other clinicopathological parameters according to the same criteria used for CLM after a washout period of at least 3 months with no special training during that time [13,27]. As for counting mitoses, the pathologists were provided instructions for annotating areas corresponding to a total area of 2.38 mm^2^, which corresponds to the area in the high-power fields evaluated using an eyepiece with a field diameter of 0.55 mm to perform MCs. ER, PR, HER2, and Ki67 IHC were also re-scored using WSI and the same CLM criteria.

### 2.4. Digital Image Analysis

**Figure 2:**
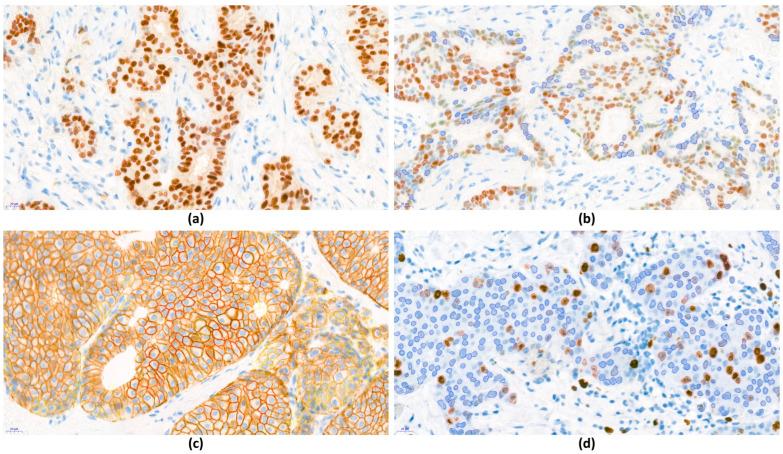
Examples of images analyzed using digital image analysis (DIA) software for the assessment of breast cancer biomarker expression (QuantCenter Digital Image Analysis Software Version 2.2, 3DHISTECH, Budapest, Hungary). (**a**) Tumor cells strongly stained for ER, detected via the software, and highlighted as red circles. (**b**) PR-stained slide from the same case as ER, exhibiting a more heterogenous pattern compared to that of ER. Different staining intensities are indicated by color (0: blue; 1+: yellow; 2+: orange; 3+: red). (**c**) HER2-stained image classified as 0 (blue), 1+ (yellow), 2+ (orange), or 3+ (red). (**d**) Ki67 staining, identified as negative (blue) or positive (red). (**a**–**d**) Images captured at original magnification: ×40.

Images of IHC stained slides from WSI were analyzed using DIA software (QuantCenter Digital Image Analysis Software Version 2.2; 3DHISTECH). Firstly, the images were reviewed by a breast pathologist at low magnification to identify and select the invasive tumor area to be scored. At least five areas to be scored were selected to represent the spectrum of staining observed in the initial WSI overview. The expression of each biomarker in the selected fields was analyzed using DIA software, applying the same scoring methods as those used by the pathologist for visual scoring, and the mean value of each case was obtained (Figure 2).

### 2.5. Definition of Perfect Concordance, Minor Discordance, and Major Discordance

Perfect concordance was established as an absolute agreement between histological grading and biomarker expression. In histological grading and its components, minor discordance was defined as a disparity between grades 1 and 2 or grades 2 and 3. A major discordance can arise when there is a grading disparity of more than one level. In IHC staining, perfect concordance for ER and PR was defined as the same Allred score being assigned. For HER2 and Ki67, perfect concordance was defined as scores of 0, 1, 2, or 3 being matched. Minor concordance was defined as different staining scores with no clinical implications. Major discordance was defined as a notable shift in staining results that could have clinical implications, including positive versus negative outcomes for ER, PR, and HER2, as well as instances of equivocal versus negative HER2 staining [4]. For Ki67, a grading discrepancy of more than one level was defined as major discordance.

### 2.6. Statistical Analysis

Cohen’s kappa was utilized to assess intra-observer agreement when comparing CLM and WSI, with higher kappa values indicating a greater level of agreement: 0.01–0.20 indicated slight, 0.21–0.40 indicated fair, 0.41–0.60 indicated moderate, 0.61–0.80 indicated substantial, and 0.81–0.99 indicated strong agreement [28]. Cohen’s kappa was also used to compare intra-observer or intra-class correlations among CLM, WSI, and DIA. The differences between CLM and WSI for histological grade and biomarker expression scores were not normally distributed (*p* for Kolmogorov–Smirnov tests < 0.01), and the Wilcoxon signed-rank test was used to compare the paired difference between CLM and WSI for all values. Fleiss’ kappa was utilized to estimate the concordance rates among the three pathologists (representing inter-observer variability) for each evaluation method. Statistical significance was set at *p* < 0.05. Statistical analyses were performed using SPSS software for Windows (version 26.0; SPSS, Chicago, IL, USA).

## 3. Results

### 3.1. Patients and Clinicopathologic Characteristics

This study included 101 cases of BC, with 46 detected in the right breast and 55 in the left breast. The diagnosed cases were histologically categorized as follows: 88 cases of invasive carcinoma of no special type, 8 cases of invasive lobular carcinoma, 2 cases of invasive mucinous carcinoma, 1 case of papillary carcinoma, 1 case of tubular carcinoma, and 1 case characterized by a mixed presentation of invasive ductal and lobular carcinoma. All patients included in this study were female, with a median age of 55 years (range, 36–88 years).

### 3.2. Intra-Observer Concordance and Agreement of Nottingham Grade and Its Components between CLM and WSI

**Figure 3:**
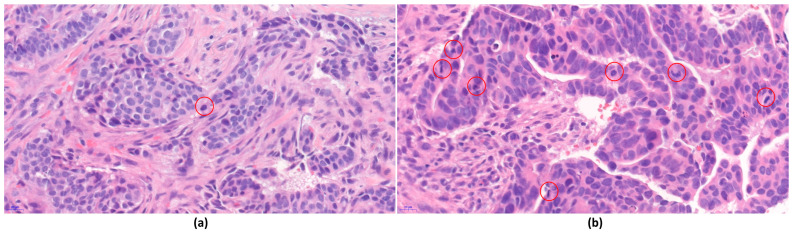
An example of minor discordant Nottingham combined histologic grade and its component scores between CLM and WSI, demonstrating both intra-observer and inter-observer discordance. The specimen comprised two biopsy cores exhibiting heterogeneous histologic patterns. (**a**) One core showed poor glandular differentiation but had lower mitotic counts. (**b**) The other core displayed enhanced glandular differentiation but had higher mitotic counts. (**a**,**b**) WSI showed the possible appearance of mitosis-like figures surrounded by red circles. The images were captured at original magnification: ×40.

Perfect concordance of NG between CLM and WSI was achieved in 78 (77.2%), 81 (80.2%), and 77 (76.2%) cases identified by the three observers (Table 1). Minor discordance was observed in 23 (22.8%), 20 (19.8%), and 24 (23.8%) cases (Figure 3). No major discordance was observed among the three observers. For the individual components of the histological grade, perfect concordance for TF was achieved in 79 (78.2%), 83 (82.2%), and 84 (83.2%) cases. Perfect concordance for NP was attained in 74 (73.3%), 78 (77.2%), and 65 (64.4%) cases. Perfect concordance of MCs was observed in 87 (86.1%), 84 (83.2%), and 84 (83.2%) cases. Although no major discordance was observed for TF and NP, four cases (3.9%) of major discordance were observed in MCs by one observer.

**Figure 4:**
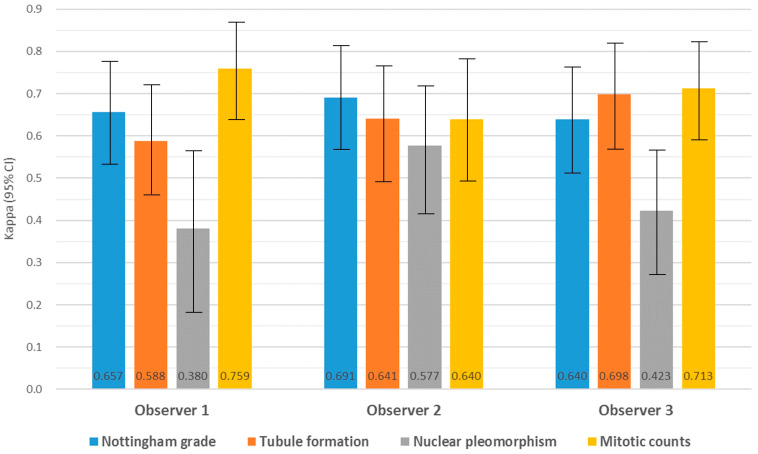
Intra-observer agreement of Nottingham combined histologic grade and its component scores between CLM and WSI utilizing kappa. All kappa coefficients demonstrated significance (*p* < 0.001).

Intra-observer agreement for NG between CLM and WSI was substantial for all observers. For the individual grade components, TF and MCs showed moderate to substantial agreement. For NP, the degree of agreement ranged from fair to moderate for all observers (Figure 4 and Table S1). There was no significant difference in the variance between the WSI and CLM agreement rates for NG and its components (all kappa coefficients showed *p* values < 0.001).

Comparing the paired difference in NG between CLM and WSI, CLM had a higher grade than WSI for one observer. For TF, one observer showed a higher score with CLM than WSI, whereas the other two observers showed lower scores with CLM than WSI. For NP, the two observers achieved higher scores with CLM than WSI (Table 2). No significant paired differences were observed in MCs using CLM or WSI among the three observers.

### 3.3. Inter-Observer Agreement for Nottingham Grade and Its Components in CLM and WSI

Inter-observer agreement for NG was substantial both in CLM and WSI. For the individual categories, the degree of agreement ranged from moderate in TF to substantial in MC and fair in NP (Table 3).

### 3.4. Agreement and Intra-Observer Variability in Biomarker Expression

Strong intra-observer concordance was observed between CLM and WSI by all three pathologists (Table 4). Perfect ER concordance for each observer was obtained in 92 (91.0%), 89 (88.1%), and 92 cases (91.1%). Perfect concordance of PR for each observer was achieved in 74 (73.2%), 66 (65.3%), and 80 (79.2%) cases. For HER2, perfect concordance was observed in 85 (84.1%), 93 (92.0%), and 80 (79.2%) cases for each observer. Ki67 staining showed perfect concordance in 86 (85.2%), 78 (77.3%), and 84 (83.2%) patients. The major discordance of HER2 was higher than those of the other biomarkers for two observers, with 11 (10.9%) and 10 cases (9.9%).

**Figure 5:**
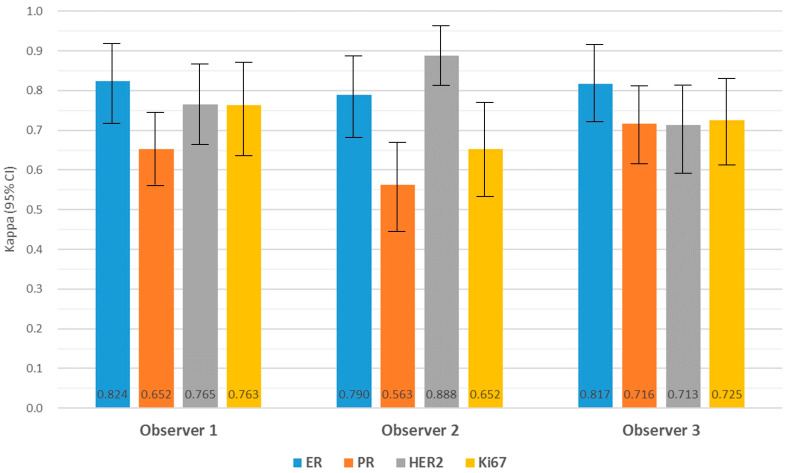
Intra-observer agreement of breast cancer biomarker expression between CLM and WSI using kappa. All kappa coefficients demonstrated significance (*p* < 0.001).

The intra-observer agreement of BC biomarker expression between CLM and WSI is shown in Figure 5 (see also Table S2). For ER, the degree of agreement ranged from substantial to strong for the three observers (κ = 0.824, κ = 0.790, and κ = 0.817). Moderate to substantial concordance was obtained in PR (κ = 0.652, κ = 0.563, and κ = 0.716). HER2 staining showed substantial to strong concordance (κ = 0.765, κ = 0.888, and κ = 0.713). In Ki67, substantial concordance was achieved (κ = 0.763, κ = 0.652, and κ = 0.725). Statistical analysis revealed no significant differences in variance between the WSI and CLM agreement rates for all four biomarkers (all kappa coefficients were *p* < 0.001). When comparing paired differences in biomarker expression between CLM and WSI, there was no clear bias in intra-observer variability in the expression of the four biomarkers among the three observers (Table 5).

### 3.5. Inter-Observer Variability in Biomarker Expression

Inter-observer agreement for ER was substantial both in CLM and WSI (Fleiss’ κ = 0.792 and Fleiss’ κ = 0.783, respectively) (Table 6). For PR, the degree of agreement was moderate to substantial (Fleiss’ κ = 0.598 in CLM and Fleiss’ κ = 0.648 in WSI). In HER2, substantial inter-observer agreement was reported in CLM and WSI (Fleiss’ κ = 0.680 in CLM and Fleiss’ κ = 0.618 in WSI). The degree of agreement for Ki67 showed Fleiss’ kappa coefficients of 0.577 for CLM and 0.642 for WSI.

### 3.6. Evaluation of Biomarker Expression with DIA

**Figure 6:**
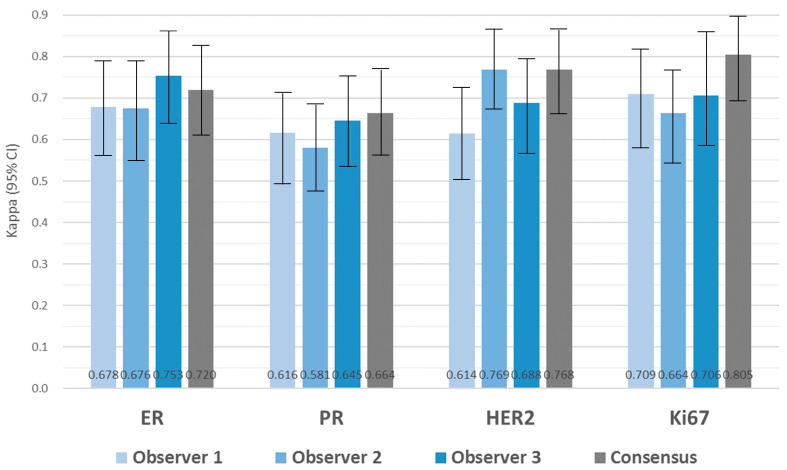
Agreement for breast cancer biomarker expression between CLM and DIA among three observers and their consensus. All kappa coefficients demonstrated significance (*p* < 0.001).

**Figure 7:**
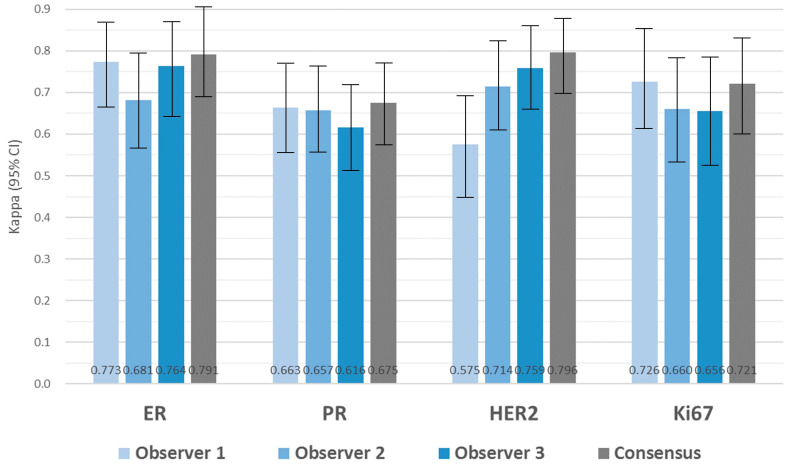
Agreement for breast cancer biomarker expression between WSI and DIA among three observers and their consensus. All kappa coefficients demonstrated significance (*p* < 0.001).

The comparison of BC biomarker expression between with CLM and DIA, as well as between WSI and DIA, was conducted for each observer. The results revealed moderate to substantial agreement among observers, with kappa values ranging from 0.676 to 0.753 for ER, 0.581 to 0.645 for PR, 0.614 to 0.769 for HER2, and 0.664 to 0.709 for Ki67 in the CLM/DIA comparison (Figure 6, Table S3). Similar kappa agreements were observed in the WSI/DIA comparison, ranging from 0.681 to 0.773 for ER, 0.616 to 0.663 for PR, 0.575 to 0.759 for HER2, and 0.656 to 0.726 for Ki67 (Figure 7, Table S3). To assess the utility of DIA, following a consensus meeting between the three observers, the intra-class correlations between CLM and DIA and between WSI and DIA were evaluated, and the results are presented in Figure 6 and Figure 7 (see also Table S4). For ER, the degree of intra-class agreement was substantial between CLM and DIA (κ = 0.720) and between WSI and DIA (κ = 0.791). PR agreement was substantial in both intra-class analyses (κ = 0.664 for CLM/DIA and κ = 0.675 for WSI/DIA). For HER2, the agreement was substantial for both the compared methods (κ = 0.768 for CLM/DIA and κ = 0.796 for WSI/DIA). Ki67 interpretation achieved substantial to strong intra-class concordance (κ = 0.805 for CLM/DIA and κ = 0.721 for WSI/DIA).

## 4. Discussion

The grading of BC using the Nottingham combined histological grade is one of the strongest prognostic factors, independent of tumor size or the number of positive lymph nodes, and it is also incorporated into the AJCC Cancer Staging Manual [29,30]. Despite increasing interest in utilizing WSI for primary diagnostic purposes, the digital validation of BC prognostic factors has not yet been established in the literature. This study achieved a substantial level of intra-observer agreement for NG and its components among three pathologists between CLM and WSI. Furthermore, the inter-observer agreement regarding NG and its associated elements in WSI displayed agreement levels similar to that in CLM, comparable to the concordance rates reported by diverse pathologists who assessed BC grading using CLM (κ = 0.48–0.70) [31,32,33].

As for the individual components of NG, intra-observer agreement for NP scores was the most variable for all three observers. Moreover, NP showed the lowest agreement rate for inter-observer comparisons with CLM and WSI. Consistently, in previous studies, NP had the lowest intra-observer agreement of all components of NG between CLM and WSI [7,34] and the worst agreement component in inter-observer variation using WSI [35,36]. As NP lacks a quantitative definition, in contrast to TF and MCs, it emerges as the least reproducible among the three grading components. Therefore, when interpretating NP, it is crucial to meticulously examine and compare it with the surrounding normal breast epithelium through not only CLM but also WSI. Compared to previous studies with no clear biases by format [7,34], in the present study, two observers showed consistently higher NP scores for CLM than for WSI, indicating bias. In contrast, for TF, two observers showed lower scores for CLM than with WSI. Additionally, previous studies have reported that WSI shows reduced ability to identify MCs [34,37]. Rakha et al. demonstrated that, among the three NG components, the most challenging to evaluate by WSI was MCs because of the difficulty in discerning mitotic figures from apoptotic cells [7]. They also recommended using a higher magnification (×40) to ensure adequate resolution for accurate grading. In the present study, we conducted WSI and graded MCs at ×40 magnification. The improvement in the MC agreement rate through high-magnification scanning is worth noting. However, the substantial size of the files may limit the utility of this technique for routine diagnostic purposes, especially considering the high storage capacity and costs involved [18]. In the present study, even though all H&E slides were derived from CNB specimens, the scanned file size was substantial (range: 0.59–4.97 GB; mean: 1.95 GB). Efforts to reduce storage requirements are necessary to make this approach more practical for diagnostic purposes.

For patients with BC, determining prognosis and treatment strategies based on ER, PR, HER2, and Ki67 status depends on accurate IHC evaluation [3,6]. The conventional approach for IHC assessment involves visually determining and scoring positivity by manually counting stained cells. Although WSI has gained broader acceptance in surgical pathology for primary diagnosis, the digital validation of BC biomarker expression has not been established [4]. Previous studies have attempted to employ WSI to validate primary diagnoses when reporting breast biomarkers; most were focused on HER2 stains, reporting a substantial kappa value (κ = 0.791) and substantial agreement percentages (range, 61.3–92.5%) [38,39]. In the present study, consistent results were observed, with a substantial level of perfect concordance (65.3–92.0%) and kappa coefficients (0.563–0.888) for the CLM/WSI pairs in evaluating ER, PR, HER2, and Ki67 expression. Based on these findings, a consensus was reached that WSI is non-inferior to CLM when interpreting breast biomarkers, although each pathologist achieved slightly different concordance rates. Furthermore, there are concerns regarding the differences in color tone and contrast of immunostained materials when scanned into the WSI device. The HER2 scores on WSI were shown to be higher than those on glass slides, possibly because of the increased color contrast in WSI [38]. The current study revealed no apparent biases regarding intra-observer variability concerning HER2 scores based on the format used. This pattern was consistently observed for other biomarkers. Including IHC-positive controls in the slides likely contributed to this consistency, as described in a previous study [4]. Additionally, PR concordance was slightly lower than that of ER. Previous studies have consistently indicated that PR expression shows lower agreement than ER expression in assessing inter-observer variability [40,41]. PR is a target gene regulated by estrogen and naturally displays greater homogeneity in normal breast tissues and tumors [22,42]. Intermediate biomarker expression categories are less reproducible than categories at the extremes [43,44]. Therefore, the heterogeneous expression of PR may be linked to reduced levels of intra-observer agreement, and a more cautious approach is advised for observers when interpreting biomarkers within tumors exhibiting heterogeneous expression, not just through CLM but also through WSI.

In clinical practice, IHC is considered a standard diagnostic tool for tumor classification, therapeutic decision-making, and prognostic factors in BC and other malignancies [5,45]. Nevertheless, manual interpretation of BC biomarker expression has inherent limitations, such as subjectivity and variability between different observers [46]. In the present study, we assessed inter-observer concordance of BC biomarker expression through visual assessment, revealing lower concordance rates, especially for PR and Ki67 using CLM and HER2 using WSI. Importantly, our findings suggest that inter-observer variability is not specific to particular biomarkers or expression patterns. Automated DIA, conversely, is a promising alternative that could produce precise results with enhanced accuracy and reliability [17,19,47]. However, a consensus statement from the College of American Pathologists expert panel underscores the necessity of validating the use of DIA against other methods, acknowledging the insufficient published data available to establish best practices [48]. In the present study, the application of DIA to assess biomarker expression exhibited an enhanced kappa coefficient compared with the inter-observer agreement, particularly for HER2 and Ki67. Notably, when compared to each observer’s individual assessment, DIA exhibited an improved kappa coefficient when considering the consensus of three observers for the expression of most biomarkers, both with CLM and WSI. This study’s results align with previous observations, suggesting that automated HER2 IHC measurements are more comparable to consensus visual scores determined by multiple pathologists, as well as HER2 gene amplification data [49]. Given the impracticality of achieving consensus scoring by experts in routine practice, DIA may enhance the quality of biomarker expression assessment. These findings highlight the capability of DIA to improve agreement and concordance in biomarker expression assessment compared to manual assessment with CLM, as well as the consistency of results with WSI. The observed agreements emphasize that integrating DIA into the diagnostic workflow in clinical practice can significantly enhance scoring reproducibility among observers and improve objective assessment.

Given the recent efforts to validate WSI, it is crucial to underscore its numerous potential benefits [50]. WSI facilitates the easy exchange in pathological opinions between medical institutions located remotely, improves pathology education and learning experiences by enhancing educational environments, can enhance the accuracy and efficiency of pathological interpretation through automated DIA and computer-aided tools, and decreases problems associated with the retrieval of glass slides from physical storage sites. However, intra-observer discrepancies remain problematic, particularly in borderline, difficult, or challenging cases, which are often sources of disagreement [16]. Difficulties in identifying mitotic figures, nuclear details, and chromatin patterns are also commonly reported [51]. Integrating DIA is useful for quantifying pathological images and identifying objects and can enhance the consistency and accuracy of pathological interpretation [17]. However, it requires technical skills for the implementation and maintenance of complex DIA software and difficulty in accurately identifying some pathological features due to limitations in algorithms.

One of the major limitations of this study was the relatively small number of cases. This study was conducted at a single institution, which could affect the external validity of the results as variations may arise in different clinical settings, with diverse devices, or based on the pathologist’s training level. Additionally, because all samples included in this study were CNB, the results may differ from those of the excision samples. However, as this study’s aim was to assess WSI’s effectiveness and reliability as a primary diagnostic tool, focusing on histological grade and the assessment of biomarker expression in BC CNB, we believe that the use of WSI could be viewed as a strength. Furthermore, this study’s data are vital for developing guidelines and protocols for integrating WSI into routine pathology practice, ultimately enhancing diagnostic accuracy.

## 5. Conclusions

Overall, the results of inter- and intra-observer agreements regarding NG and its components, along with the assessment of biomarker expression in BCs, indicated no significant difference between the interpretations from CLM and WSI. However, a more cautious approach is advisable when interpreting histological grading and biomarker expression within tumors exhibiting heterogeneous histological or biomarker expression patterns. This study offers substantial evidence supporting the use of WSI for assessing prognostic and predictive variables in BC, including NG and biomarker expression, for routine diagnostic purposes. Furthermore, the incorporation of DIA for assessing biomarker expression has the potential to significantly improve scoring reproducibility.

## Figures and Tables

**Table 1:** Intra-observer concordance of the Nottingham combined histologic grade and its component scores between CLM and WSI.

	Perfect Concordance	Minor Discordance	Major Discordance
Observer 1			
Nottingham grade	78 (77.2%)	23 (22.8%)	0 (0.0%)
Tubule formation	79 (78.2%)	22 (21.8%)	0 (0.0%)
Nuclear pleomorphism	74 (73.3%)	27 (27.7%)	0 (0.0%)
Mitotic counts	87 (86.1%)	14 (13.9%)	0 (0.0%)
Observer 2			
Nottingham grade	81 (80.2%)	20 (19.8%)	0 (0.0%)
Tubule formation	83 (82.2%)	18 (17.8%)	0 (0.0%)
Nuclear pleomorphism	78 (77.2%)	23 (22.8%)	0 (0.0%)
Mitotic counts	84 (83.2%)	13 (12.9%)	4 (3.9%)
Observer 3			
Nottingham grade	77 (76.2%)	24 (23.8%)	0 (0.0%)
Tubule formation	84 (83.2%)	17 (16.8%)	0 (0.0%)
Nuclear pleomorphism	65 (64.4%)	36 (35.6%)	0 (0.0%)
Mitotic counts	84 (83.2%)	17 (16.8%)	0 (0.0%)

CLM, conventional light microscopy; WSI, whole slide imaging.

**Table 2:** Results of Wilcoxon signed-rank test comparing Nottingham combined histologic grade and its component scores between CLM and WSI for each observer.

	Observer 1		Observer 2		Observer 3	
	Z-Score	*p*-Value	Z-Score	*p*	Z-Score	*p*-Value
Nottingham grade	−2.294	**0.022**	−1.342	0.180	−0.816	0.541
Tubule formation	−4.264	**<0.001**	−3.771	**<0.001**	−3.638	**<0.001**
Nuclear pleomorphism	−0.557	0.564	−3.545	**<0.001**	−2.667	0.008
Mitotic counts	0.000	1.000	−0.876	0.381	−1.231	0.225

CLM, conventional light microscopy; WSI, whole slide imaging. *p* values in bold indicate significance (*p* < 0.05).

**Table 3:** Inter-observer agreement of Nottingham combined histologic grade and its component scores in CLM and WSI.

	CLM		WSI	
	Fleiss Kappa (95% CI)	*p*-Value	Fleiss Kappa (95% CI)	*p*-Value
Nottingham grade	0.630 (0.628–0.633)	**<0.001**	0.620 (0.618–0.623)	**<0.001**
Tubule formation	0.543 (0.540–0.546)	**<0.001**	0.523 (0.519–0.526)	**<0.001**
Nuclear pleomorphism	0.356 (0.353–0.359)	**<0.001**	0.394 (0.391–0.397)	**<0.001**
Mitotic counts	0.654 (0.651–0.657)	**<0.001**	0.720 (0.717–0.723)	**<0.001**

CLM, conventional light microscopy; WSI, whole slide imaging; CI, confidence interval. *p* values in bold indicate significance (*p* < 0.05).

**Table 4:** Intra-observer concordance of breast cancer biomarker expression between CLM and WSI.

	Perfect Concordance	Minor Discordance	Major Discordance
Observer 1			
ER	92 (91.0%)	5 (5.0%)	4 (4.0%)
PR	74 (73.2%)	23 (22.8%)	4 (4.0%)
HER2	85 (84.1%)	5 (5.0%)	11 (10.9%)
Ki67	86 (85.2%)	15 (14.8%)	0 (0.0%)
Observer 2			
ER	89 (88.1%)	9 (8.9%)	3 (3.0%)
PR	66 (65.3%)	32 (31.7%)	3 (3.0%)
HER2	93 (92.0%)	6 (6.0%)	2 (2.0%)
Ki67	78 (77.3%)	23 (22.7%)	0 (0.0%)
Observer 3			
ER	92 (91.1%)	8 (7.9%)	1 (1.0%)
PR	80 (79.2%)	19 (18.8%)	2 (2.0%)
HER2	80 (79.2%)	11 (10.9%)	10 (9.9%)
Ki67	84 (83.2%)	17 (16.8%)	0 (0.0%)

CLM, conventional light microscopy; WSI, whole slide imaging; CI, confidence interval.

**Table 5:** Results of Wilcoxon signed-rank test comparing breast cancer biomarker expression between CLM and WSI for each observer.

	Observer 1		Observer 2		Observer 3	
	Z-Score	*p*-Value	Z-Score	*p*	Z-Score	*p*-Value
ER	−0.420	0.674	−0.243	0.808	−0.490	0.624
PR	−0.596	0.551	−1.553	0.120	−0.600	0.549
HER2	−0.688	0.491	0.000	1.000	−1.528	0.127
Ki67	−0.258	0.796	−1.877	0.061	−1.698	0.090

CLM, conventional light microscopy; WSI, whole slide imaging.

**Table 6:** Inter-observer agreement of breast cancer biomarker expression in CLM and WSI.

	CLM		WSI	
	Fleiss Kappa (95% CI)	*p*-Value	Fleiss Kappa (95% CI)	*p*-Value
ER	0.792 (0.790–0.795)	**<0.001**	0.783 (0.781–0.786)	**<0.001**
PR	0.598 (0.596–0.600)	**<0.001**	0.648 (0.646–0.650)	**<0.001**
HER2	0.680 (0.678–0.683)	**<0.001**	0.618 (0.615–0.620)	**<0.001**
Ki67	0.577 (0.575–0.580)	**<0.001**	0.642 (0.639–0.644)	**<0.001**

CLM, conventional light microscopy; WSI, whole slide imaging; CI, confidence interval. *p* values in bold indicate significance (*p* < 0.05).

## Data Availability

The datasets generated and/or analyzed during the current study are available from the corresponding author on reasonable request.

## Supplementary Materials

The following supporting information can be downloaded via this link: https://www.mdpi.com/article/10.3390/jpm14030312/s1, Table S1: Intra-observer agreement of the Nottingham combined histologic grade and its component scores between CLM and WSI utilizing kappa; Table S2: Intra-observer agreement of the breast cancer biomarker expression between CLM and WSI utilizing kappa; Table S3: The agreement of breast cancer biomarker expression among three observers between CLM and DIA and between WSI and DIA; Table S4: Intra-class agreement of breast cancer biomarker expression between CLM and DIA and between WSI and DIA.

## References

[1] Giuliano A.E., Connolly J.L., Edge S.B., Mittendorf E.A., Rugo H.S., Solin L.J., Weaver D.L., Winchester D.J., Hortobagyi G.N. (2017). Breast Cancer-Major changes in the American Joint Committee on Cancer eighth edition cancer staging manual. CA Cancer J. Clin..

[2] Allison K.H. (2018). Ancillary Prognostic and Predictive Testing in Breast Cancer: Focus on Discordant, Unusual, and Borderline Results. Surg. Pathol. Clin..

[3] Nielsen T.O., Leung S.C.Y., Rimm D.L., Dodson A., Acs B., Badve S., Denkert C., Ellis M.J., Fineberg S., Flowers M. (2021). Assessment of Ki67 in Breast Cancer: Updated Recommendations From the International Ki67 in Breast Cancer Working Group. J. Natl. Cancer Inst..

[4] Salama A.M., Hanna M.G., Giri D., Kezlarian B., Jean M.H., Lin O., Vallejo C., Brogi E., Edelweiss M. (2022). Digital validation of breast biomarkers (ER, PR, AR, and HER2) in cytology specimens using three different scanners. Mod. Pathol. Off. J. United States Can. Acad. Pathol. Inc..

[5] Cuzick J., Dowsett M., Pineda S., Wale C., Salter J., Quinn E., Zabaglo L., Mallon E., Green A.R., Ellis I.O. (2011). Prognostic value of a combined estrogen receptor, progesterone receptor, Ki-67, and human epidermal growth factor receptor 2 immunohistochemical score and comparison with the Genomic Health recurrence score in early breast cancer. J. Clin. Oncol..

[6] Goldhirsch A., Wood W.C., Coates A.S., Gelber R.D., Thurlimann B., Senn H.J., Panel m. (2011). Strategies for subtypes—Dealing with the diversity of breast cancer: Highlights of the St. Gallen International Expert Consensus on the Primary Therapy of Early Breast Cancer 2011. Ann. Oncol..

[7] Rakha E.A., Aleskandarani M., Toss M.S., Green A.R., Ball G., Ellis I.O., Dalton L.W. (2018). Breast cancer histologic grading using digital microscopy: Concordance and outcome association. J. Clin. Pathol..

[8] Weinstein R.S., Holcomb M.J., Krupinski E.A. (2019). Invention and Early History of Telepathology (1985–2000). J. Pathol. Inform..

[9] Williams B.J., Lee J., Oien K.A., Treanor D. (2018). Digital pathology access and usage in the UK: Results from a national survey on behalf of the National Cancer Research Institute’s CM-Path initiative. J. Clin. Pathol..

[10] Pantanowitz L., Sinard J.H., Henricks W.H., Fatheree L.A., Carter A.B., Contis L., Beckwith B.A., Evans A.J., Lal A., Parwani A.V. (2013). Validating whole slide imaging for diagnostic purposes in pathology: Guideline from the College of American Pathologists Pathology and Laboratory Quality Center. Arch. Pathol. Lab. Med..

[11] Cornish T.C., Swapp R.E., Kaplan K.J. (2012). Whole-slide imaging: Routine pathologic diagnosis. Adv. Anat. Pathol..

[12] Lange H. (2011). Digital Pathology: A Regulatory Overview. Lab. Med..

[13] Evans A.J., Brown R.W., Bui M.M., Chlipala E.A., Lacchetti C., Milner D.A., Pantanowitz L., Parwani A.V., Reid K., Riben M.W. (2022). Validating Whole Slide Imaging Systems for Diagnostic Purposes in Pathology. Arch. Pathol. Lab. Med..

[14] Reyes C., Ikpatt O.F., Nadji M., Cote R.J. (2014). Intra-observer reproducibility of whole slide imaging for the primary diagnosis of breast needle biopsies. J. Pathol. Inform..

[15] Krishnamurthy S., Mathews K., McClure S., Murray M., Gilcrease M., Albarracin C., Spinosa J., Chang B., Ho J., Holt J. (2013). Multi-institutional comparison of whole slide digital imaging and optical microscopy for interpretation of hematoxylin-eosin-stained breast tissue sections. Arch. Pathol. Lab. Med..

[16] Araújo A.L.D., Arboleda L.P.A., Palmier N.R., Fonsêca J.M., de Pauli Paglioni M., Gomes-Silva W., Ribeiro A.C.P., Brandão T.B., Simonato L.E., Speight P.M. (2019). The performance of digital microscopy for primary diagnosis in human pathology: A systematic review. Virchows Arch..

[17] Stålhammar G., Fuentes Martinez N., Lippert M., Tobin N.P., Mølholm I., Kis L., Rosin G., Rantalainen M., Pedersen L., Bergh J. (2016). Digital image analysis outperforms manual biomarker assessment in breast cancer. Mod. Pathol. Off. J. United States Can. Acad. Pathol. Inc..

[18] Al-Janabi S., Huisman A., Van Diest P.J. (2012). Digital pathology: Current status and future perspectives. Histopathology.

[19] Yousif M., Huang Y., Sciallis A., Kleer C.G., Pang J., Smola B., Naik K., McClintock D.S., Zhao L., Kunju L.P. (2022). Quantitative Image Analysis as an Adjunct to Manual Scoring of ER, PgR, and HER2 in Invasive Breast Carcinoma. Am. J. Clin. Pathol..

[20] Di Loreto C., Puglisi F., Rimondi G., Zuiani C., Anania G., Della Mea V., Beltrami C.A. (1996). Large core biopsy for diagnostic and prognostic evaluation of invasive breast carcinomas. Eur. J. Cancer.

[21] Rakha E.A., Allison K.H., Ellis I.O., Penault-Llorca F., Vincent-Salomon A., Masuda S., Tsuda H., Horii R., Allison K.H., Salgado R. (2019). Invasive breast carcinoma: General overview. WHO Classification of Tumours. Breast Tumours.

[22] Rossi C., Fraticelli S., Fanizza M., Ferrari A., Ferraris E., Messina A., Della Valle A., Anghelone C.A.P., Lasagna A., Rizzo G. (2023). Concordance of immunohistochemistry for predictive and prognostic factors in breast cancer between biopsy and surgical excision: A single-centre experience and review of the literature. Breast Cancer Res. Treat..

[23] Hammond M.E., Hayes D.F., Dowsett M., Allred D.C., Hagerty K.L., Badve S., Fitzgibbons P.L., Francis G., Goldstein N.S., Hayes M. (2010). American Society of Clinical Oncology/College Of American Pathologists guideline recommendations for immunohistochemical testing of estrogen and progesterone receptors in breast cancer. J. Clin. Oncol..

[24] Elston C.W., Ellis I.O. (1991). Pathological prognostic factors in breast cancer. I. The value of histological grade in breast cancer: Experience from a large study with long-term follow-up. Histopathology.

[25] Allred D.C., Harvey J.M., Berardo M., Clark G.M. (1998). Prognostic and predictive factors in breast cancer by immunohistochemical analysis. Mod. Pathol. Off. J. United States Can. Acad. Pathol. Inc..

[26] Wolff A.C., Hammond M.E.H., Allison K.H., Harvey B.E., Mangu P.B., Bartlett J.M.S., Bilous M., Ellis I.O., Fitzgibbons P., Hanna W. (2018). Human Epidermal Growth Factor Receptor 2 Testing in Breast Cancer: American Society of Clinical Oncology/College of American Pathologists Clinical Practice Guideline Focused Update. Arch. Pathol. Lab. Med..

[27] Chong Y., Kim D.C., Jung C.K., Kim D.C., Song S.Y., Joo H.J., Yi S.Y. (2020). Recommendations for pathologic practice using digital pathology: Consensus report of the Korean Society of Pathologists. J. Pathol. Transl. Med..

[28] Landis J.R., Koch G.G. (1977). The measurement of observer agreement for categorical data. Biometrics.

[29] Hortobagyi G.N., Connolly J.L., D’Orsi C.J., Edge S.B., Mittendorf E.A., Rugo H.S., Solin L.J., Weaver D.L., Winchester D.J., Giuliano A., American Joint Committee on Cancer (2017). AJCC Cancer Staging Manual 8th edition. American Joint Committee on Cancer.

[30] Schwartz A.M., Henson D.E., Chen D., Rajamarthandan S. (2014). Histologic grade remains a prognostic factor for breast cancer regardless of the number of positive lymph nodes and tumor size: A study of 161 708 cases of breast cancer from the SEER Program. Arch. Pathol. Lab. Med..

[31] Meyer J.S., Alvarez C., Milikowski C., Olson N., Russo I., Russo J., Glass A., Zehnbauer B.A., Lister K., Parwaresch R. (2005). Breast carcinoma malignancy grading by Bloom-Richardson system vs proliferation index: Reproducibility of grade and advantages of proliferation index. Mod. Pathol. Off. J. United States Can. Acad. Pathol. Inc..

[32] Longacre T.A., Ennis M., Quenneville L.A., Bane A.L., Bleiweiss I.J., Carter B.A., Catelano E., Hendrickson M.R., Hibshoosh H., Layfield L.J. (2006). Interobserver agreement and reproducibility in classification of invasive breast carcinoma: An NCI breast cancer family registry study. Mod. Pathol. Off. J. United States Can. Acad. Pathol. Inc..

[33] Rakha E.A., Bennett R.L., Coleman D., Pinder S.E., Ellis I.O. (2017). Review of the national external quality assessment (EQA) scheme for breast pathology in the UK. J. Clin. Pathol..

[34] Davidson T.M., Rendi M.H., Frederick P.D., Onega T., Allison K.H., Mercan E., Brunyé T.T., Shapiro L.G., Weaver D.L., Elmore J.G. (2019). Breast Cancer Prognostic Factors in the Digital Era: Comparison of Nottingham Grade using Whole Slide Images and Glass Slides. J. Pathol. Inform..

[35] Ginter P.S., Idress R., D’Alfonso T.M., Fineberg S., Jaffer S., Sattar A.K., Chagpar A., Wilson P., Harigopal M. (2021). Histologic grading of breast carcinoma: A multi-institution study of interobserver variation using virtual microscopy. Mod. Pathol. Off. J. United States Can. Acad. Pathol. Inc..

[36] Shaw E.C., Hanby A.M., Wheeler K., Shaaban A.M., Poller D., Barton S., Treanor D., Fulford L., Walker R.A., Ryan D. (2012). Observer agreement comparing the use of virtual slides with glass slides in the pathology review component of the POSH breast cancer cohort study. J. Clin. Pathol..

[37] Rakha E.A., Aleskandarany M.A., Toss M.S., Mongan N.P., ElSayed M.E., Green A.R., Ellis I.O., Dalton L.W. (2018). Impact of breast cancer grade discordance on prediction of outcome. Histopathology.

[38] Kondo Y., Iijima T., Noguchi M. (2012). Evaluation of immunohistochemical staining using whole-slide imaging for HER2 scoring of breast cancer in comparison with real glass slides. Pathol. Int..

[39] Nassar A., Cohen C., Albitar M., Agersborg S.S., Zhou W., Lynch K.A., Heyman E.R., Lange H., Siddiqui M.T. (2011). Reading immunohistochemical slides on a computer monitor—A multisite performance study using 180 HER2-stained breast carcinomas. Appl. Immunohistochem. Mol. Morphol..

[40] Cserni B., Bori R., Csörgő E., Oláh-Németh O., Pancsa T., Sejben A., Sejben I., Vörös A., Zombori T., Nyári T. (2021). The additional value of ONEST (Observers Needed to Evaluate Subjective Tests) in assessing reproducibility of oestrogen receptor, progesterone receptor, and Ki67 classification in breast cancer. Virchows Arch..

[41] Regitnig P., Reiner A., Dinges H.P., Höfler G., Müller-Holzner E., Lax S.F., Obrist P., Rudas M., Quehenberger F. (2002). Quality assurance for detection of estrogen and progesterone receptors by immunohistochemistry in Austrian pathology laboratories. Virchows Arch..

[42] Baird R.D., Carroll J.S. (2016). Understanding Oestrogen Receptor Function in Breast Cancer and its Interaction with the Progesterone Receptor. New Preclinical Findings and their Clinical Implications. Clin. Oncol. (R. Coll. Radiol.).

[43] Wells C.A., Sloane J.P., Coleman D., Munt C., Amendoeira I., Apostolikas N., Bellocq J.P., Bianchi S., Boecker W., Bussolati G. (2004). Consistency of staining and reporting of oestrogen receptor immunocytochemistry within the European Union--an inter-laboratory study. Virchows Arch..

[44] Pu T., Shui R., Shi J., Liang Z., Yang W., Bu H., Li Q., Zhang Z. (2019). External quality assessment (EQA) program for the immunohistochemical detection of ER, PR and Ki-67 in breast cancer: Results of an interlaboratory reproducibility ring study in China. BMC Cancer.

[45] Inamura K. (2018). Update on Immunohistochemistry for the Diagnosis of Lung Cancer. Cancers.

[46] Stålhammar G., Rosin G., Fredriksson I., Bergh J., Hartman J. (2014). Low concordance of biomarkers in histopathological and cytological material from breast cancer. Histopathology.

[47] Bera K., Schalper K.A., Rimm D.L., Velcheti V., Madabhushi A. (2019). Artificial intelligence in digital pathology—New tools for diagnosis and precision oncology. Nat. Rev. Clin. Oncol..

[48] Bui M.M., Riben M.W., Allison K.H., Chlipala E., Colasacco C., Kahn A.G., Lacchetti C., Madabhushi A., Pantanowitz L., Salama M.E. (2019). Quantitative Image Analysis of Human Epidermal Growth Factor Receptor 2 Immunohistochemistry for Breast Cancer: Guideline From the College of American Pathologists. Arch. Pathol. Lab. Med..

[49] Skaland I., Ovestad I., Janssen E.A., Klos J., Kjellevold K.H., Helliesen T., Baak J.P. (2008). Digital image analysis improves the quality of subjective HER-2 expression scoring in breast cancer. Appl. Immunohistochem. Mol. Morphol..

[50] Mukhopadhyay S., Feldman M.D., Abels E., Ashfaq R., Beltaifa S., Cacciabeve N.G., Cathro H.P., Cheng L., Cooper K., Dickey G.E. (2018). Whole Slide Imaging Versus Microscopy for Primary Diagnosis in Surgical Pathology: A Multicenter Blinded Randomized Noninferiority Study of 1992 Cases (Pivotal Study). Am. J. Surg. Pathol..

[51] Pekmezci M., Uysal S.P., Orhan Y., Tihan T., Lee H.S. (2016). Pitfalls in the use of whole slide imaging for the diagnosis of central nervous system tumors: A pilot study in surgical neuropathology. J. Pathol. Inform..

