# Supplementary material for: Digital Validation in Breast Cancer Needle Biopsies: Comparison of Histological Grade and Biomarker Expression Assessment Using Conventional Light Microscopy, Whole Slide Imaging, and Digital Image Analysis
Source: J Pers Med. 2024 Mar 16;14(3):312. doi: 10.3390/jpm14030312 (PMC10971358; doi:10.3390/jpm14030312)
Supplement: Supplementary file 1 [file jpm-14-00312-s001.zip › jpm-2885653-supplementary.pdf]

Table S1. Intra-observer agreement of the Nottingham combined histologic grade and its component scores between CLM and WSI utilizing kappa.

	**Observer 1**	**Observer 2**	**Observer 3**
	**Kappa (95% CI)**	**Kappa (95% CI)**	**Kappa (95% CI)**
Nottingham grade	0.657 (0.537–0.776)	0.691 (0.566–0.815)	0.640 (0.507–0.761)
Tubule formation	0.588 (0.459–0.722)	0.641 (0.494–0.772)	0.698 (0.568–0.821)
Nuclear pleomorphism	0.380 (0.181–0.563)	0.577 (0.417–0.720)	0.423 (0.267–0.564)
Mitotic counts	0.759 (0.640–0.866)	0.640 (0.494–0.783)	0.713 (0.595–0.825)

CLM, conventional light microscopy; WSI, whole slide imaging; CI, confidence interval. All kappa coefficients demonstrated significance ($p < 0.001$).

Table S2. Intra-observer agreement of the breast cancer biomarker expression between CLM and WSI utilizing kappa.

	**Observer 1**	**Observer 2**	**Observer 3**
	**Kappa (95% CI)**	**Kappa (95% CI)**	**Kappa (95% CI)**
ER	0.824 (0.712–0.919)	0.790 (0.683–0.888)	0.817 (0.717–0.911)
PR	0.652 (0.555–0.745)	0.563 (0.449–0.670)	0.716 (0.612–0.805)
HER2	0.765 (0.657–0.866)	0.888 (0.809–0.957)	0.713 (0.591–0.812)
Ki67	0.763 (0.639–0.872)	0.652 (0.532–0.771)	0.725 (0.606–0.836)

CLM, conventional light microscopy; WSI, whole slide imaging; CI, confidence interval; ER, estrogen receptor; PR, progesterone receptor; HER2, human epidermal receptor 2. All kappa coefficients demonstrated significance ($p < 0.001$).

Table S3. The agreement of breast cancer biomarker expression among three observers between CLM and DIA, and between WSI and DIA.

	**CLM / DIA**		**WSI / DIA**	
	**Kappa (95% CI)**	***p*-value**	**Kappa (95% CI)**	***p*-value**
**Observer 1**				
ER	0.678 (0.555–0.789)	**< 0.001**	0.773 (0.662–0.868)	**< 0.001**
PR	0.616 (0.496–0.716)	**< 0.001**	0.663 (0.551–0.768)	**< 0.001**
HER2	0.614 (0.500–0.725)	**< 0.001**	0.575 (0.449–0.691)	**< 0.001**
Ki67	0.709 (0.576–0.821)	**< 0.001**	0.726 (0.610–0.851)	**< 0.001**
**Observer 2**				
ER	0.676 (0.549–0.790)	**< 0.001**	0.681 (0.566–0.794)	**< 0.001**
PR	0.581 (0.475–0.684)	**< 0.001**	0.657 (0.553–0.759)	**< 0.001**
HER2	0.769 (0.668–0.863)	**< 0.001**	0.714 (0.605–0.823)	**< 0.001**
Ki67	0.664 (0.546–0.772)	**< 0.001**	0.660 (0.536–0.783)	**< 0.001**
**Observer 3**				
ER	0.753 (0.639–0.861)	**< 0.001**	0.764 (0.643–0.872)	**< 0.001**
PR	0.645 (0.538–0.752)	**< 0.001**	0.616 (0.510–0.719)	**< 0.001**
HER2	0.688 (0.565–0.794)	**< 0.001**	0.759 (0.655–0.857)	**< 0.001**
Ki67	0.706 (0.584–0.855)	**< 0.001**	0.656 (0.526–0.786)	**< 0.001**

CLM, conventional light microscopy; DIA, digital image analysis; WSI, whole slide imaging; CI, confidence interval; ER, estrogen receptor; PR, progesterone receptor; HER2, human epidermal receptor 2. *P* values in bold indicate significance ($p < 0.05$).

Table S4. Intra-class agreement of breast cancer biomarker expression between CLM and DIA, and between WSI and DIA.

	**CLM / DIA**		**WSI / DIA**	
	**Kappa (95% CI)**	***p*-value**	**Kappa (95% CI)**	***p*-value**
ER	0.720 (0.606–0.825)	**< 0.001**	0.791 (0.690–0.903)	**< 0.001**
PR	0.664 (0.565–0.769)	**< 0.001**	0.675 (0.571–0.772)	**< 0.001**
HER2	0.768 (0.655–0.863)	**< 0.001**	0.796 (0.698–0.878)	**< 0.001**
Ki67	0.805 (0.694–0.899)	**< 0.001**	0.721 (0.600–0.833)	**< 0.001**

CLM, conventional light microscopy; DIA, digital image analysis; WSI, whole slide imaging; CI, confidence interval; ER, estrogen receptor; PR, progesterone receptor; HER2, human epidermal receptor 2. *P* values in bold indicate significance ($p < 0.05$).
